# Psychometric validation of the Turkish expanded mindful eating scale

**DOI:** 10.1371/journal.pone.0328175

**Published:** 2025-07-16

**Authors:** Gökcen Doğan, Eda Çakmak, Ceren Şarahman Kahraman, Nurcan Yabanci Ayhan

**Affiliations:** 1 Health Sciences Faculty, Department of Nutrition and Dietetics, Lokman Hekim University, Ankara, Turkey; 2 Biostatistics, Health Sciences Faculty, Department of Audiology, Başkent University, Ankara, Turkey; 3 Health Sciences Faculty, Department of Nutrition and Dietetics, Alanya Alaaddin Keykubat Üniversitesi, Antalya, Turkey; 4 Health Sciences Faculty, Department of Nutrition and Dietetics, Ankara University, Ankara, Turkey; Necmettin Erbakan Üniversitesi: Necmettin Erbakan Universitesi, TÜRKIYE

## Abstract

**Background/Objectives:**

The Expanded Mindful Eating Scale (EMES) assesses mindfulness by considering both individual health and environmental sustainability. A distinguishing feature of the EMES is its inclusion of sustainability, setting it apart from other eating awareness scales. This study was carried out to determine the validity and reliability of the Turkish culture-adapted version of the EMES in adults.

**Materials/Methods:**

In this cross-sectional study, 662 Turkish adults (mean age=29.8, SD=12.18; 18.4% male) were recruited via an online questionnaire. In this study, participants were recruited via an online survey that included their sociodemographic characteristics, the EMES, Mindful Eating Questionnaire (MEQ), and sick, control, one, fat, food (SCOFF) Eating Disorders Scale. Confirmatory factor analysis (CFA) was used to test the validity of the EMES. Analyses were conducted using SPSS version 25.0 and SPSS AMOS version 25.0. The 5-factor structure identified by Explanatory Factor Analysis (EFA) was validated using CFA.

**Results:**

As a result of confirmatory factor analysis (RMSEA: 0.053, CFI: 0.89, GFI: 0.94, and AGFI: 0.92), model fit indices were obtained within acceptable limits. In the reliability analysis, it was observed that the EMES scale had a good level of reliability with the Spearman-Brown coefficient. A significant correlation was observed between the EMES and MEQ.

**Conclusion:**

The results showed that the use of the Turkish version of the EMES is valid and reliable. It is thought that the validation of the Expanded Mindful Eating Scale reflects a comprehensive approach that recognizes the interconnectedness of individual health and environmental well-being.

## Introduction

Obesity is a major obstacle to the quality of life all over the world and has a great effect on the increase in the frequency of chronic diseases [1]. According to the Turkish Nutrition and Health Survey, which was completed with the participation of 24,000 people in Turkey, the prevalence of obesity in male and female individuals over 19 years old is 24.9% and 29.2%, respectively. Morbid obesity has been reported as 1.4% and 7.0% [2]. Turkey has the highest prevalence in the European region [3]. It is known that the frequency of maladaptive eating behaviors (e.g., external eating, emotional eating and eating to cope with negative feelings) is high in obese individuals [4]. In recent years, researchers and practitioners have focused on “mindful eating” to prevent maladaptive eating behaviors [5]. Mindful eating comes from mindfulness practices based on Buddhist teachings. It was first used as a therapy or intervention in an 8-week stress management program developed by Kabat-Zinn in the 1970s [6]. Later, mindfulness was used in cognitive therapy methods for use in the treatment of anxiety and depression. Today, this form of cognitive therapy is endorsed by the UK National Institute for Health and Care Excellence (NICE) [7].

Mindful eating is a practice that focuses on enjoying food through all the senses, promoting present-moment awareness and an attitude of nonjudgmental attention. It involves a heightened sense of consciousness and deliberate focus on the sensory aspects of food consumption, including taste, texture, and smell [8,9]. By cultivating mindfulness during meals, individuals can develop a greater understanding of their internal cues of hunger and fullness, as well as the psychological factors that influence their eating behaviors [10]. Additionally, mindful eating can promote healthier eating habits, weight management, and overall well-being. By fostering a nonreactive and nonjudgmental attitude toward food, individuals can enhance their ability to make conscious choices and develop a more positive relationship with eating. Moreover, practicing mindful eating has been associated with reduced emotional eating, external eating, and binge eating. As a result, incorporating mindful eating techniques into interventions and programs aimed at improving nutrition and addressing disordered eating patterns may hold promise for enhancing individuals’ overall health and psychological well-being [11–13]. Additionally, mindfulness interventions have been recommended by healthcare guidelines for addressing maladaptive eating behaviors and improving overall health [14].

An important feature that distinguishes the Expanded Mindful Eating Scale from other eating awareness scales is that it includes sustainability. It tries to raise awareness by simultaneously considering the health of both individuals and the planet [15,16]. It is also known that Buddhist approaches, which form the basis for the emergence of eating awareness, include approaches to protect the health of not only the individual but also the entire planet, living or non-living [17]. By integrating sustainability into mindful eating practices, individuals are prompted to consider the broader impact of their food choices on personal health and environmental sustainability [18].

The incorporation of sustainability into the Expanded Mindful Eating Scale reflects a comprehensive approach that recognizes the interconnectedness of individual health and environmental well-being. By promoting mindfulness in food choices that consider sustainability, this scale contributes to a more holistic understanding of health that goes beyond individual benefits to encompass broader ecological considerations. In this study, it was aimed to adapt the Expanded Mindful Eating Scale (EMES) to Turkish culture in a sample of adults.

## Materials and methods

### Adaptation protocol

Initially, permission to conduct the study was obtained via email from the developers of the scale [16]. Following the process of cross-cultural adaptation developed by Beaton et al. (2020) [19], the Turkish version of the scale was created. In this stage, five independent translators were involved. The translated versions were assessed by Nutrition and Dietetics experts for consistency and semantic accuracy, and the scale adaptation process was completed following the required revisions. Before proceeding to the data collection phase, a pilot study involving 40 participants was conducted to assess the comprehensibility of the scale items. The data collected during the pilot study were excluded from the study’s final analysis.

### Participants

The study was conducted with the participation of a total of 662 adult individuals residing in Turkey, with a mean age of 29.81 ± 12.18 years, and 18.4% (n = 122) of the participants were male. The data was obtained between January/2023 and January/2025. In adaptation studies, it is recommended to achieve a sample size that is 5–10 times the number of items on the scale. The study data were collected online using Google Forms. Individuals were reached through social media platforms (WhatsApp, Facebook, Instagram, etc.) to participate in the survey. This study was conducted according to the guidelines laid down in the Declaration of Helsinki and all procedures involving research study participants were approved by Ankara University, Rectorate Research Ethics Committee (approval number: 02/24/410983).

### Measures

To evaluate the study objectives, a comprehensive set of validated instruments was utilized. These tools were selected based on their relevance to assessing mindful eating, disordered eating behaviors, and related psychological factors in a Turkish-speaking population.

### Questionnaire form

A written informed consent section was placed at the beginning of the questionnaire. Following this section, participants gave their consent in the form of a question by choosing “I approve to participate” or “I do not approve”. The questionnaire form consists of four sections. The first section contains questions about general information, including age, gender, and education. Participants self-reported their body weight and height, and body mass index (BMI) was calculated by dividing weight in kilograms by the square of height in meters [20]. The second and third sections of the survey include the EMES and the Mindful Eating Questionnaire (MEQ), respectively. The final section contains SCOFF Eating Disorders Scale.

### Expanded mindful eating scale

The EMES, developed by Kawasaki et al., consists of a final factor structure with five factors and 20 items, identified through Explanatory Factor Analysis (EFA) [16]. The scale employs a four-point Likert-type rating system, ranging from 1 (disagree) to 4 (agree). The EMES contains five sub-factors (Health of the planet/ Factor 1, Awareness and appreciation for food/ Factor 2, Nonreactivity/ Factor 3, Non-judgmental awareness/ Factor 4, and Hunger and satiety cues/ Factor 5). An increase in scores on the scale indicates an increase in an individual’s level of mindful eating awareness. Using CFA, the authors reported the following fit indices for the final model: Goodness of Fit Index (GFI) = 0.914, Adjusted Goodness of Fit Index (AGFI) = 0.890, Comparative Fit Index (CFI) = 0.870, and Root Mean Square Error of Approximation (RMSEA) = 0.061. Kawasaki et al. observed significant correlations between the total EMES score and BMI, mindfulness, body dissatisfaction, drive for thinness, and life satisfaction (r = –0.138, –0.315, –0.339, –0.281, and 0.149, respectively; p < 0.01). The Cronbach’s alpha for all items on the scale was 0.687 [16].

### Mindful eating questionnaire

The MEQ, developed by Benitez et al. (2009) [21], was adapted into Turkish in 2016 [22]. The MEQ factor analysis revealed seven underlying factors: Disinhibition, Emotional Eating, Control of Eating, Eating Discipline, Mindfulness, Interference. Based on expert recommendations, non-functioning items were removed, new items were added to the valid items, and the scale was adapted as the Mindful Eating Scale (MES-30), consisting of 30 questions. The adapted scale utilized a 5-point Likert scale (1: never, 2: rarely, 3: sometimes, 4: often, 5: always). The Cronbach alpha value for the MES-30 scale was 0.733 [23].

### SCOFF eating disorders scale

The SCOFF Eating Disorders Scale, developed by Morgan, Reid, and Lacey in 1999, was designed to identify eating disorder cases at the primary care level [24]. The scale was designed with a small number of screening items to screen for eating disorders and provide a prompt for further examination and research. Aydemir et al. conducted the validity and reliability study of the scale in Turkish in 2015 [23]. The scale comprises five questions, each with two possible answers. Each item is scored as 1, and individuals with a total score of 2 or higher are classified as being at risk for eating disorders [18].

### Statistical analysis

Statistical analyses were performed using the Statistical Package for the Social Sciences (SPSS) version 25.0 and SPSS AMOS version 25.0 (IBM Corp., Armonk, NY USA). The reliability of the EMES was evaluated using the Cronbach’s alpha coefficient and the Spearman-Brown coefficient. The validity of the scale was assessed through explanatory and confirmatory factor analyses. The suitability of the sample for factor analysis was tested using the Kaiser-Meyer-Olkin (KMO) measure of sample adequacy. The principal component method with direct oblimin rotation was applied in the EFA, and the Kaiser criterion was used to determine the number of factors. Confirmatory factor analysis (CFA) was conducted to evaluate the suitability of the identified factor structure. Model fit indices were assessed using RMSEA, CFI, and GFI. The Kolmogorov-Smirnov test was employed to evaluate the normality of the distribution, while the Levene test assessed variance homogeneity. For comparisons between two groups, the Mann-Whitney U test was utilized, and differences among three groups were analyzed using the Bonferroni Corrected Mann-Whitney U test. The correlation between the scales was calculated with the Spearman Rank Correlation Coefficient. For all analyses, a p-value of <0.05 was considered statistically significant.

## Results

The comparative findings of the EMES scores for the demographic data of the study are summarized in Table 1.

**Table 1:** Characteristics of the study population.

	Mean ± SD (Min–Max)
**Age (years)**	29.81 ± 12.18 (18–65)
Male	34.85 ± 13.92 (18–65)
Female	28.67 ± 11.47 (18–65)
**Body weight (kg)**	64.43 ± 13.58 (37–120)
Male	78.83 ± 12.89 (52–120)
Female	61.18 ± 11.46 (37–120)
**Height (cm)**	166.17 ± 8.34 (140–191)
Male	176.53 ± 7.39 (155–191)
Female	163.83 ± 6.59 (140–185)
	**n (%)**
**Gender**	
Male	122 (18.4%)
Female	540 (81.6%)
**Marital status**	
Single	460 (69.5%)
Married	202 (30.5%)
**Nutritional status**	
Underweight (BMI < 18.5)	56 (8.5%)
Normal weight (18.5 ≤ BMI < 25.0)	418 (63.1%)
Pre-obesity (25.0 ≤ BMI < 30.0)	141 (21.3%)
Obesity (BMI > 30.0)	47 (7.1%)
**Educational Status**	
Non-educated	22 (3.3%)
Primary school	22 (3.3%)
High school	208 (31.4%)
University	410 (61.9%)
**Place of residence**	
With family	423 (63.9%)
With friends	53 (8%)
Alone	70 (10.6%)
Dormitory	116 (17.5%)
**Use of cigarettes**	
Ex-smoker	37 (5.6%)
Yes	168 (25.4%)
No	457 (69%)
**Use of alcohol**	
Ex-drinker	13 (2%)
Yes	231 (34.9%)
No	418 (63.1%)
**Body weight satisfaction**	
I wish I was heavier	101 (15.3%)
I wish I was weaker	322 (48.6%)
I am satisfied	239 (36.1%)

### Validity analysis

#### Explanatory factor analysis.

For the EFA of the scale, the adequacy of the sample and the suitability of the analysis were tested. It was significant with the Bartlett test of sphericity (*χ2* = 2703.11, df = 190, P < 0.001), and the Kaiser-Meyer-Olkin (KMO) coefficient of 0.78 was sufficient for the sample. An examination of the diagonal values of the inverse correlation matrix for the EMES revealed no evidence of multicollinearity among the variables.

Principal component analysis with the varimax rotation method were used to determine the factor structure of the scale. Five factors according to a Kaiser criterion greater than 1 eigenvalue were obtained, and the total variance explanation percentage of the scale was calculated as 52.26%. The variance explanation percentages of these factors were obtained as Factor 1 (18.23%), Factor 2 (13.13%), Factor 3 (8.21%), Factor 4 (6.95%), and Factor 5 (5.74%), respectively. Information about the factors and the factor loads obtained is presented in Table 2.

**Table 2:** EMES EFA results.

EMES items	Factor 1	Factor 2	Factor 3	Factor 4	Factor 5
**Factor 1**	(Eigen value: 3.646, Variance (%): 18.229, Cronbach alpha: 0.773)				
Q3	0.762				
Q4	0.734				
Q5	0.781				
Q6	0.695				
**Factor 2**	(Eigen value: 2.626, Variance (%): 13.132, Cronbach alpha: 0.677)				
Q15		0.760			
Q16		0.733			
Q18		0.557			
Q19		0.554			
Q20		0.528			
**Factor 3**	(Eigen value: 1.641, Variance (%): 8.206, Cronbach alpha: 0.657)				
Q8			0.657		
Q9			0.669		
Q10			0.470		
Q11			0.543		
Q17			0.696		
**Factor 4**	(Eigen value:1.390, Variance (%): 6.951, Cronbach alpha: 0.482)				
Q12		0.417		0.150	
Q13				0.835	
Q14				0.843	
**Factor 5**	(Eigen value: 1.149, Variance (%): 5.744, Cronbach alpha: 0.559)				
Q1					0.646
Q2					0.752
Q7					0.427

#### Confirmatory factor analysis.

**Fig 1:**
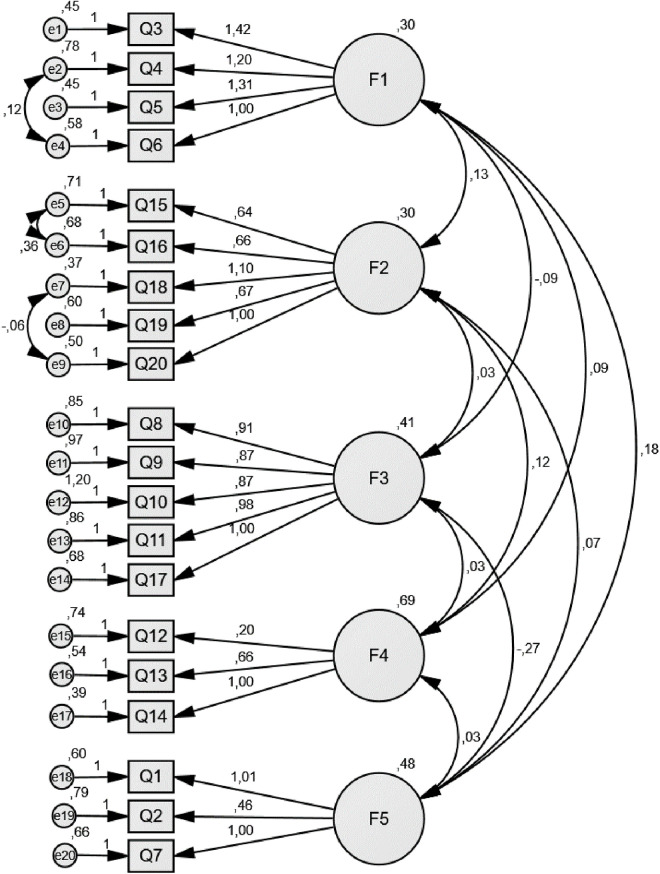
Confirmatory factor analysis.

CFA was used to validate the 5-factor structure explained by EFA and the model is given in the Fig 1. Model fit indices *χ2*/df: 2.83, RMSEA: 0.053, CFI: 0.89, GFI: 0.94, and AGFI: 0.92, were obtained with CFA and were observed to be within acceptable limits.

### Reliability analysis

In the reliability analysis of the EMES, the Cronbach alpha coefficient for the whole scale was 0.64 and the sub-factors were calculated as 0.77 for Factor 1, 0.68 for Factor 2, 0.66 for Factor 3, 0.48 for Factor 4, and 0.56 for Factor 5, respectively. As a result of the split half method, the Spearman-Brown reliability coefficient was obtained as 0.75.

A significant correlation (*r*_*s*_ = 0.55, P < 0.05) was observed between the EMES and MEQ regarding the total score, and the correlation values for the sub-factors are given in Table 3.

**Table 3:** EMES total score, mean and SD values of the factors, and the relationship between them.

	Mean ± SD	EMES Total	EMES F1	EMES F2	EMES F3	EMES F4	EMES F5
**EMES total**	51.17 ± 9.16	–	0.517**	0.754**	0.550**	0.045	0.486**
**F1**	11.01 ± 3.14		–	0.165**	0.081*	–0.213**	0.336**
**F2**	13.35 ± 6.07			–	0.133**	–0.125**	0.060
**F3**	13.11 ± 3.25				–	0.024	0.306**
**F4**	5.03 ± 1.99					–	−0.099*
**F5**	8.67 ± 2.23						–
**MEQ total**	96.93 ± 15.89	0.554*					
**F1**	15.62 ± 4.92	0.443**	0.161**	0.102**	0.585**	0.025	0.441**
**F2**	15.81 ± 5.49	0.421**	0.150**	0.144**	0.573**	–0.057	0.344**
**F3**	14.33 ± 3.90	0.298**	0.192**	0.061	0.337**	–0.020	0.312**
**F4**	16.11 ± 2.05	0.156**	0.162**	0.129**	0.045	–0.074	0.064
**F5**	12.91 ± 3.19	0.431**	0.386**	0.292**	0.222*	–0.203**	0.292**
**F6**	15.13 ± 2.87	0.250**	0.235**	0.042	0.255**	0.005	0.207**
**F7**	7.01 ± 1.92	0.382**	0.227**	0.126**	0.454**	−0.025	0.266**

Pearson correlation, *P < 0.05, ** P < 0.001

According to the SCOFF Scale, a difference was observed in terms of the EMES and MEQ total scores of individuals with and without an eating disorder risk. In addition, the difference for the sub-factors was also examined and is summarized in Table 4.

**Table 4:** Investigation of the EMES and MEQ scale according to the SCOFF scale.

	SCOFF		
	No risk for an eating disorder (n = 428)	At risk for an eating disorder (n = 234)	
	Mean ± SD (min–max)	Mean ± SD (min–max)	P-value
**EMES Total**	53.03 ± 8.59 (27–73)	47.77 ± 9.23 (21–68)	<0.001^U^
**F1.**	11.23 ± 3.11 (4–16)	10.62 ± 3.17 (4–16)	0.027^U^
**F2.**	13.88 ± 5.79 (1–20)	12.36 ± 6.45 (1–20)	0.005^U^
**F3.**	13.79 ± 3.12 (5–20)	11.85 ± 3.12 (5–20)	<0.001^U^
**F4.**	4.99 ± 1.96 (3–10)	5.10 ± 2.04 (3–12)	0.524^U^
**F5.**	9.14 ± 2.06 (3–12)	7.82 ± 2.29 (3–12)	<0.001^U^
**MEQ Total**	101.12 ± 14.49 (52–138)	89.28 ± 15.52 (50–138)	<0.001^U^
**F1.**	16.85 ± 4.53 (5–25)	13.38 ± 4.81 (5–25)	<0.001^U^
**F2.**	17.16 ± 4.99 (5–25)	13.33 ± 5.50 (5–25)	<0.001^U^
**F3.**	15 ± 3.74 (4–20)	13.11 ± 3.89 (4–20)	<0.001^U^
**F4.**	16.19 ± 2.01 (10–23)	15.97 ± 2.12 (7–22)	0.106^U^
**F5.**	13.21 ± 3.10 (4–20)	12.36 ± 3.28 (4–20)	0.001^U^
**F6.**	15.44 ± 2.89 (8–25)	14.56 ± 2.74 (5–22)	0.001^U^
**F7.**	7.25 ± 1.91 (2–10)	6.58 ± 1.87 (2–10)	<0.001^U^

U: Mann-Whitney U test.

## Discussion

The current study aimed to examine the psychometric characteristics of the Turkish adaptation of the EMES in an adult population. This study constitutes the first validation attempt of the scale following its translation into Turkish. The Turkish version of the scale was administered online to adults, and data from 662 participants were analyzed.

In this study, the scale’s 5-factor structure and 20 questions remained the same as in the original scale. CFA confirmed the model fit, which was found to be within acceptable limits. In the reliability analysis of the EMES, the Cronbach alpha coefficient for the whole scale was 0.64 and the sub-factors were calculated as 0.77 for Factor 1, 0.68 for Factor 2, 0.66 for Factor 3, 0.48 for Factor 4, and 0.56 for factor 5, respectively. Although Item 12 yielded a higher Cronbach’s alpha value when loaded onto Factor 2, it was retained under Factor 4 due to its thematic relevance. Furthermore, as this is the first validation study of EMES in a non-English language, preserving the original factorial integrity was prioritized to allow for future cross-cultural comparisons and longitudinal validation studies. In the present study, some subscales, particularly Factor 4 (Non-judgmental awareness), showed relatively low internal consistency and weak or non-significant correlations with other subscales and related constructs. This may suggest that Factor 4 captures a distinct dimension of mindful eating that is not strongly aligned with the constructs measured by the MEQ subscales. All of these results showed that the Turkish version of the EMES is a reliable screening scale.

Developed by Kawasaki et al. in 2020 [16], the EMES has not yet been validated in any other language. However, in this study, it showed a statistically significant positive correlation with the total score of MEQ and its seven underlying factors (Disinhibition, Emotional Eating, Control of Eating, Eating Discipline, Mindfulness, Conscious nutrition, Interference), which has been used in Turkey since 2016. Unlike the MEQ, EMES includes items on environmental friendliness and animal welfare in Factor 1, offering a broader, ecologically-informed perspective on mindful eating. In addition, an appreciation for food is emphasized under factor 2. In Turkey, where no existing scale addresses issues related to environmental friendliness and animal welfare in the context of mindful eating, the EMES fills a crucial gap by introducing these important considerations. By including factors that promote awareness of the broader implications of food choices, the EMES offers a comprehensive tool for individuals to cultivate a mindful eating practice that not only benefits their own health but also contributes to the well-being of the planet [25].

Mindful eating is one of the popular methods used in the treatment of eating disorders, emotional eating, and obesity in recent years [26–28]. In this study, there was a significant difference in terms of the EMES scores between individuals at risk and not at risk for an eating disorder according to the SCOFF Scale. Individuals at risk of eating disorders have been shown to have lower EMES scores. These findings were similar to different mindful eating scales in the literature [29,30].

Mindful eating has emerged as a significant intervention for addressing eating disorders, emotional eating, and obesity, gaining traction in recent years as a viable therapeutic approach. Research indicates that mindful eating practices can enhance awareness of eating-related emotions and habits, fostering a non-judgmental observation of thoughts and feelings associated with food consumption [31]. This awareness is crucial in the prevention and treatment of eating behavior disorders, as it allows individuals to recognize and modify maladaptive eating patterns without the pressure of self-judgment [32–34]. Mindfulness-based interventions can lead to improved emotional regulation and healthier eating behaviors among those at risk for eating disorders. This is particularly relevant due to the high prevalence of emotional eating among individuals with obesity, in whom stress and negative emotions frequently trigger maladaptive eating behaviors [35–37]. Mindful eating constitutes a transformative approach that not only addresses immediate emotional and unhealthy eating patterns but also contributes to sustainable lifestyle changes. This is imperative in a society where the prevalence of eating disorders and obesity continues to rise, making mindful eating a valuable tool for clinicians and individuals looking to improve their relationship with food [38]. Emphasizing self-compassion and awareness within eating processes could thus present a paradigm shift in treating not just eating disorders but also fostering healthy lifestyle choices among various populations.

The study has several limitations that should be considered. Although participants of both genders were included, the sample had a significantly higher proportion of females, which may limit the generalizability of the findings. Additionally, data collection through online surveys introduced potential selection bias, with participants having higher education levels than the national average. The reliance on self-reported data for variables such as body weight and height may have led to measurement inaccuracies. Furthermore, the risk of eating disorders was assessed using the SCOFF scale rather than clinical diagnoses, which may have affected the accuracy of group classifications. Despite these limitations, the study also has notable strengths. It is the first to adapt and validate the Expanded Mindful Eating Scale (EMES) for a Turkish-speaking population, providing a significant contribution to the field. The EMES offers a unique perspective by incorporating sustainability-related factors, bridging individual health with environmental well-being. The study employed rigorous statistical analyses, including explanatory and confirmatory factor analyses, to ensure robust psychometric evaluation. Additionally, the large sample size, which adhered to recommended guidelines, enhances the reliability of the findings. By following established cross-cultural adaptation protocols, the study ensured the scale’s cultural and semantic relevance to the target population.

The results obtained in this study showed that the use of the Turkish version of the EMES is valid and reliable. It is thought that the use of this scale, which will increase awareness on sustainable issues such as Health of the planet and Appreciation for food, will make a great contribution to the field in this developing country, where the concept of food awareness is newly learned. In future studies, it is predicted that studying the EMES on topics such as food label reading habits and health literacy will make a wide contribution and different perspective to the field.

## Supporting information

S1 File English Version of the Scale. (DOCX)

S2 File Turkish Version of the Scale. (DOCX)

S1 Appendix The scoring of the scale and sub-factors. (DOCX)
