# Supplementary material for: Psychometric validation of the Turkish expanded mindful eating scale
Source: PLoS One. 2025 Jul 16;20(7):e0328175. doi: 10.1371/journal.pone.0328175 (PMC12266424; doi:10.1371/journal.pone.0328175)
Supplement: S1 File — (DOCX) [file pone.0328175.s001.docx]

|  | **The Expanded Mindful Eating Scale** |  |
| --- | --- | --- |
| 1 | I end the meal once I feel I have eaten the right amount. | Factor 5: Hunger and satiety cues |
| 2 | I eat meals to match my hunger. | Factor 5: Hunger and satiety cues |
| 3 | I buy foods that are environmentally friendly, such as those that have no (or fewer) pesticides, and no (or low) chemicals. | Factor 1/ Health of the planet |
| 4 | I buy meat products that are animal welfare friendly, such as pasture-raised meat and free-range eggs. | Factor 1/ Health of the planet |
| 5 | I buy food products that have less packaging and are environmentally friendly. | Factor 1/ Health of the planet |
| 6 | I buy meals and prepared food that make use of local ingredients or regional products. | Factor 1/ Health of the planet |
| 7 | When I feel that I may end up overeating, I do not reach for more food at once; I take a little bit of rest before reaching for more food. | Factor 5: Hunger and satiety cues |
| 8* | When I see an advertisement for a food product that looks tempting, I buy that product without hesitation even if there is a chance that I might overeat. | Factor 3: Nonreactivity |
| 9* | When my stomach is empty, all I tend to think about is food. | Factor 3: Nonreactivity |
| 10* | I eat too much when I am stressed or anxious about something. | Factor 3: Nonreactivity |
| 11* | When I eat out or go to an all-you-can-eat buffet, I overeat. | Factor 3: Nonreactivity |
| 12* | I can tell if my daily meal is the right or wrong meal. | Factor 4: Non-judgmental awareness |
| 13* | There is good food and bad food. | Factor 4: Non-judgmental awareness |
| 14* | It is not good to want to eat food that may be bad for the health. | Factor 4: Non-judgmental awareness |
| 15 | When I eat food, I savor each and every bite. | Factor 2: Awareness and appreciation for food |
| 16 | I eat my meals while enjoying their appearance, color and aroma. | Factor 2: Awareness and appreciation for food |
| 17* | I unconsciously eat meals or snacks. | Factor 3: Nonreactivity |
| 18 | I savor the changing of the seasons in the food ingredients and dishes that I eat. | Factor 2: Awareness and appreciation for food |
| 19 | I enjoy the meals offered in seasonal celebrations such as the New Year and Hinamatsuri (Doll’s Festival). | Factor 2: Awareness and appreciation for food |
| 20 | I am thankful for the people and materials involved in all the processes that go into creating my meals, such as the ingredients, manufacturers, cooks, etc. | Factor 2: Awareness and appreciation for food |
