# Supplementary material for: Psychometric validation of the Turkish expanded mindful eating scale
Source: PLoS One. 2025 Jul 16;20(7):e0328175. doi: 10.1371/journal.pone.0328175 (PMC12266424; doi:10.1371/journal.pone.0328175)
Supplement: S2 File — (DOCX) [file pone.0328175.s002.docx]

**APPENDIX**

The scale is a four-point Likert type and the answers are; disagree (1), somewhat disagree (2), somewhat agree (3), I agree (4).

|  | **Genişletilmiş Yeme Farkındalığı Ölçeği / The Turkish Version of The Expanded Mindful Eating Scale** |  |
| --- | --- | --- |
| 1 | Yemeyi, doğru miktarda yediğimi hissettiğimde bırakırım. | Factor 5 |
| 2 | Açlık durumuma göre yemek yerim. | Factor 5 |
| 3 | Pestisit ve kimyasal içermeyen (ya da daha düşük miktarda içeren) çevre dostu besinler satın alırım. | Factor 1 |
| 4 | Gezen tavuk yumurtası ve otlakta yetiştirilen hayvanların etleri gibi hayvan dostu et ürünleri alırım. | Factor 1 |
| 5 | Ambalajı daha az ve çevre dostu besinleri satın alırım. | Factor 1 |
| 6 | Yerel içeriklere sahip veya bölgesel ürünlerden hazırlanmış besinleri /yemekleri tercih ederim. | Factor 1 |
| 7 | Aşırı yemek yiyebileceğimi hissettiğimde, aynı anda daha fazla yiyeceğe uzanmam; daha fazla yiyeceğe ulaşmadan önce biraz dinlenirim. | Factor 5 |
| 8* | Cazip görünen bir besin reklamı gördüğümde, fazla yeme ihtimalim olsa bile tereddüt etmeden o ürünü satın alırım. | Factor 3 |
| 9* | Midem boşken, tek düşündüğüm şey yemektir. | Factor 3 |
| 10* | Bir şey hakkında stresli veya endişeli olduğumda çok fazla yerim. | Factor 3 |
| 11* | Dışarıda ya da açık büfe seçenekli bir yerde yemek yediğimde fazla yerim. | Factor 3 |
| 12* | Öğünlerimin doğru ya da yanlış olup olmadığını anlayabilirim. | Factor 4 |
| 13* | İyi besin ve kötü besin vardır. | Factor 4 |
| 14* | Sağlığa zararlı olabilecek yiyecekleri yemek istemek iyi değildir. | Factor 4 |
| 15 | Yemek yerken, her bir lokmanın tadını çıkarırım. | Factor 2 |
| 16 | Yemeklerimi görünüşünün, renginin ve aromasının tadını çıkararak yerim. | Factor 2 |
| 17* | Öğün veya atıştırmalıkları bilinçsizce tüketirim. | Factor 3 |
| 18 | Yediğim yemek ve içeriklerindeki sezonluk (mevsimsel) değişimlerden zevk alırım. | Factor 2 |
| 19 | Kandil, bayram, yeni yıl gibi özel günlerde sunulan yemeklerden hoşlanırım. | Factor 2 |
| 20 | Yemeklerimi oluşturan süreçlerde yer alan besinler, üreticiler, aşçılar gibi tüm insanlara ve malzemelere minnettarım. | Factor 2 |

The total score varies between 20-80. Score range for each factor respectively; 4-16, 5-20, 5-20, 3-12 and 3-12.

*Inversed items; 8-9-10-11-12-13-14-17
